# Reducing the pressures of outpatient care: the potential role of patient-reported outcomes

**DOI:** 10.1177/01410768231152222

**Published:** 2023-02-09

**Authors:** Olalekan Lee Aiyegbusi, Sarah E Hughes, John Devin Peipert, Liv Marit Valen Schougaard, Roger Wilson, Melanie J Calvert

**Affiliations:** 1 Institute of Applied Health Research, University of Birmingham, Birmingham, B15 2TT, UK; 2 National Institute for Health Research (NIHR) Applied Research Collaboration West Midlands, Birmingham, B15 2TT, UK; 3 NIHR Birmingham Biomedical Research Centre, University of Birmingham, Birmingham, B15 2TT, UK; 4 Birmingham Health Partners Centre for Regulatory Science and Innovation, University of Birmingham, Birmingham, B15 2TT, UK; 5 NIHR Oxford-Birmingham Blood and Transplant Research Unit (BTRU) in Precision Therapeutics, University of Birmingham, Birmingham, B15 2TT, UK; 6 Department of Medical Social Sciences, Northwestern University, Feinberg School of Medicine, Chicago, IL, 60611, USA; 7 AmbuFlex/WestChronic, Occupational Medicine, University Research Clinic, Aarhus University, 7400, Herning, Denmark; 8 NIHR Surgical Reconstruction and Microbiology Research Centre, University of Birmingham, Birmingham, B15 2TT, UK

**Keywords:** Effectiveness of care, long-term care, quality improvement, Patient-reported outcomes, ePROs, outpatient care

## Abstract

The global demand for hospital treatment exceeds capacity.The COVID-19 pandemic has exacerbated this issue, leading to increased backlogs and longer wait times for patients. The amount of outpatient attendances undertaken in many settings is still below pre-pandemic levels and this, combined with delayed referrals, means that patients are facing delays in treatment and poorer health outcomes. Use of digital health technologies, notably the use of remote symptom monitoring systems based on patient-reported outcomes (PROs), may offer a solution to reduce outpatient waiting lists and tailor care to those in greatest need. Drawing on international examples, the authors explore the use of electronic PRO systems to triage clinical care. We summarise the key benefits of the approach and also highlight the challenges for implementation, which need to be addressed to promote equitable healthcare delivery.

One way to address hospital outpatient pressures might be the effective ‘triaging’ of patients so that only those who really need to be seen in person at clinics are given face-to-face appointments. Since the beginning of the COVID-19 pandemic, telehealth has been increasingly deployed for the purpose of remotely monitoring and screening patients. One form of telehealth that may be beneficial in the outpatient setting is the use of electronic patient-reported outcome (ePRO) systems to capture patients’ symptoms, functioning and experience with treatments. PROs have been defined as ‘… any report of the status of a patient’s health condition that comes directly from the patient, without interpretation of the patient’s response by a clinician or anyone else’.^1^ While the collection and use of PROs is well established in research settings to evaluate the effectiveness, cost-effectiveness and tolerability of interventions from a patient perspective,^2^ in recent years, interest in the routine use of PROs to enhance the quality of patient care has increased and clinicians are more interested in patient-level PRO data for the clinical management of individual patients in routine practice.^2–4^ The technological innovations that have led to a rapid adoption and increased ownership of electronic devices have also facilitated the development and dissemination of ePROs, which allow patients to be sent ePRO questionnaires to fill out at home or at a location of their choice and have the results sent back to the clinician in near real time to use in clinical decision-making.^5^ Various studies have demonstrated that the collection of ePROs in routine clinical practice is both acceptable and feasible, with patients increasingly expressing a preference for an electronic mode of administration.^6,7^

In this article, we offer an international perspective on the potential benefits of using ePRO solutions to support the management of outpatient care. We summarise the benefits reported by publications on existing ePRO systems on clinical workflow and patient care. We consider and propose solutions to address barriers to implementation and use.

## Methods

### Search strategy

For this narrative review, we searched PubMed from inception on 4 July 2022, for articles that described the use of ePRO solutions in outpatient care using the words ‘clinic’, ‘outpatient’, ‘electronic patient reported outcomes’, ‘ePROs’ as search terms. We also conducted hand searching of reference lists of selected articles. Two researchers independently performed the screening of titles and abstracts and evaluation of full texts. They discussed and agreed on the included articles.

## Eligibility criteria

We included studies in English language that reported specific benefits or outcomes of using ePRO solutions in outpatient settings. We excluded narrative or systematic reviews, commentaries, opinion pieces and letters that do not report primary findings.

## Search results

Our database search retrieved 135 entries that were independently screened by OLA and MJC. The full texts for 41 articles were examined and 16 were selected. The 25 articles excluded did not meet our eligibility criteria. These either focused on the development of ePRO systems or the use of ePROs in other settings such as in clinical trials. Two more articles were identified through hand searching, bringing the total number of included articles to 18.

## Benefits of implementing ePRO systems for outpatient care

There were several reported benefits of implementing ePRO systems for outpatient care. When ePROs were administered remotely, it facilitated the triaging of patients. Symptom support was provided to clinically stable patients who did not require outpatient appointments, while patients who needed to be seen in person were given appointments and seen more quickly. This led to significant reductions in outpatient appointments and the timely provision of interventions in several clinical specialties. The administration of ePRO before clinic appointments and the discussion of ePRO results with patients during their appointment fostered a better understanding and documentation of patient symptoms, timely initiation of interventions and enhanced patient–clinician interactions. Evidence was provided by studies conducted with various groups of patients including those with epilepsy, sleep apnoea, type 1 diabetes, cancer, rheumatoid arthritis (RA) and HIV. The ePROs for most of the studies were provided remotely (14 of 16 studies), while only two studies gave patients the option of providing remotely or in clinic. The use of reminders to patients to provide their ePROs was reported by 8 (50%) of the studies, while patient training was reported by 3 (19%) and clinical staff training by 5 (31%). Key findings from the studies are provided below. Table 1 provides the details of the administration of the ePRO systems used by the studies while Table 2 summarises the study characteristics and findings.

**Table 1.** Administration of ePROs in the studies reviewed.

Study	Mode	Location	Frequency of assessments	Reminders	Duration of follow-up	Translations	Proxy completion	Training	Implementation challenges
Appel et al.^8^	Web-based	Remotely	2–12 months based on patient characteristics, disease activity, treatment, experiences, preferences Changeable over time	Reminders and an alert were sent if patients did not respond	12 months	Not reported	Patients were not enrolled if they were unable to read or fill out the PRO Questionnaire	Not reported	Not reported
Basch et al.^9^	Mobile responsive web-based interface or automated telephone system	Remotely	Weekly	Prompts and reminders were sent to patients	12 months or until treatment discontinuation	Not reported	Not reported	Yes, patients	• Variation in individual patient’s compliance with weekly reporting, ranging from 17% to 100%
de Jong et al.^10^	Web-based for tablets or smartphones	Remotely	The monitoring modules were completed monthly but when in remission, patients were allowed to complete once every 3 months	Not reported	12 months	Not reported	Not reported	Not reported	Not reported
de Thurah et al.^11^	Web-based	Remotely	3–4 months	Not reported	12 months	Not reported	Not reported	Not reported	Not reported
Efficace et al.^12^	Web-based	Remotely	Time-points were not pre-planned for the completion of PRO assessments	Patients received automated reminders every 2 weeks after first assessment	Approximately 6 months	Not reported	Not reported	Yes, clinical staff	Not reported
Girgis et al.^13^	On an iPad, in clinic	Initially in clinic, then option of in clinic or remotely	Monthly but dependent on cancer stage, treatment plan and personal preferences	Not reported	5 months	English-only assessments at the time of implementation	Patients from target group were not onboarded unless they were assisted by family or interpreters	Yes, clinical staff	• Implementation sites had large non-English-speaking populations• Almost half (46%) of the target population were ineligible due to lower levels of literacy, predominantly due to language
Haverman et al.^14^	Web-based	Remotely	Not reported	Not reported	9 months	Not reported	Proxy measures available for parents of younger children	Yes, clinical staff	Not reported
Laurberg et al.^15^	Web-based	Remotely	Every 4 months	Reminders were sent	15 months	Not reported	Not reported	Not reported	• Data collection concerning healthcare services was inaccurate, due to inconsistencies in the manner different healthcare providers registered contacts to the hospital
Richter et al.^16^	Initial completion in clinic either with a smartphone (BYOD) or paper Subsequently, both options were available remotely	In clinic initially then remotely	Every 3 months depending on their individually scheduled routine outpatient visits at clinic	Not reported	9 months	Not reported	Staff assistance in clinic on demand	Yes, patients	Not reported
Riis et al.^17^	Web-based	Remotely	Every 3 months	Reminders were sent approximately after 7 days, 14 days and 21 days	24 months	Not reported	Not reported	Not reported	Not reported
Samuel et al.^18^	Web-based or automated telephone	Remotely or in clinic	Baseline, 1 month, 3 months following treatment initiation	Not reported	3 months	Not reported	Not reported	Yes, patients	• Lower levels of computer and health literacy among the Black participants
Schick-Makaroff et al.^19^	Tablets	In clinic	Every 3 months	Not reported	6 months	Not reported	Not reported	Not reported	Not reported
Schougaard et al.^20^	Web or paper-based	Remotely	Routine use	Reminders were sent	Not relevant, as system has been implemented for routine use	Not reported	Not reported	Not reported	Not reported
Schougaard et al.^21^	Web-based	Remotely	In standard telePRO, patients filled in fixed-interval disease-specific questionnaires every 3, 6, or 12 months In open access telePRO, the patients could indicate a need for contact with the outpatient clinic by filling in the disease-specific questionnaire at any time	Reminders were sent	18 months	Not reported	Not reported	Not reported	• Activity in terms of number of logins to the ‘My Epilepsy’ web site and questionnaires filled in initiated by the patients decreased during the follow-up period• An increased number of reminders were sent to patients; the response rate (37%) was, however, low• At the organisational level, much effort was put into developing the intervention, but the implementation strategy was probably insufficient, leading to issues related to knowledge and confidence in using the intervention as intended by the patients• At the individual patient level, the open access intervention demanded some self-management skills because the patients were expected to actively interact with the healthcare system
Seppen et al.^22^	Mobile devices (Smartphones)	Remotely	Weekly	Smart phone app-based reminders to complete questionnaires were planned but a technical malfunction caused very few to be sent Automated email reminders were later sent	12 months	Not reported	Not reported	Yes, patients. The first login was done in clinic and patients were shown the features of the app	• During the trial, 24 bug reports were made regarding eight different bugs in the application. Most importantly, notifications did not work between July 2019 and February 2021 for most patients. Therefore, most patients received few or no App-based reminders during the study. Automated email reminders were later sent to minimise the impact of this bug. The adherence rate during the email reminders was similar (62%; 1009 of 1625) to that prior to the email reminders (58%; 537 of 923)
Seppen et al.^23^	Web-based	Remotely	1–2 times	Not reported	3 months	Not reported	Not reported	Not reported	Not reported
Short et al.^24^	Tablet	In clinic	Where possible, skip logic was applied to reduce respondent burden Eligibility for future follow-up assessments within the PRO platform was set for a minimum of 105 days, to minimise potential clinic burden and/or patient survey fatigue	Not reported	24 months	English, Spanish, and/or Haitian Creole	Not reported	Yes, staff training	Not reported
Zhang et al.^25^	Mobile devices or computers	Remotely	Weekly	Reminders were sent	6 months	Not reported	Inclusion criteria mentioned that caregivers could assist	Yes, staff training	• Some patients’ adherence decreased over time

PRO: patient-reported outcome.

**Table 2.** Study characteristics and findings.

Study	Setting, country	Design	Patient group	ePROM intervention, sample size (n)	Usual care (control), sample size (n)	Outcome(s) of interest	Intended use/clinical response to ePROM data	Result(s)
Appel et al.^8^	Outpatient, Denmark	Before and after cohort study (AmbuIBD) at an outpatient clinic	Patients with inflammatory bowel disease (IBD)	Of 848 patients in outpatient care, 77% (539) were included in AmbuIBD. Data analyses were done 12 months before and 12 months after participation	Not applicable	Effect of AmbuIBD on outpatient visits and hospital admissions	PRO-based telemedicine follow-up. Outpatient clinic management Dashboard presents graphical overview to inform patient–clinician communication Based on results of a decision algorithm, nurses decided whether patients need no contact, a phone call or clinic visit	• 66% of 1913 answered questionnaires were handled with no further contact.• Outpatient visits the year after AmbuIBD compared to the year before were reduced with 14% (*p* < 0.001).• Largest reduction was for patients with mild or no disease activity (45%, *p* < 0.001).• Telephone consultations decreased by 17% (*p* < 0.002) the year after inclusion in AmbuIBD.• No difference was found in hospital admissions significant for patients with mild disease (*p* = 0.014).• No difference in missed appointments (*p* < 0.88).
Basch et al.^9^	Community oncology practices, USA	PRO-TECT is a multicentre trial. Feedback surveys were administered to participating patients and clinicians	Adult patients with advanced and metastatic cancers	496 patients from 26 practices participated. Items from PRO-CTCAE were remotely administered to patients	Not applicable	User feedback	Change on PRO-CTCAE scores triggers self-management information to be sent to patients, and alerts were sent to nurses for severe or worsening symptoms over a 1-year period or till treatment was discontinued There were no requirements for the actions nurses or oncologists should take based on the ePRO or symptom management pathway information received	• 78.6% of nurses stated that ePRO data was helpful for documentation in the electronic medical record• 345/493 (70.0%) patients stated that clinicians used ePRO data at 3 months, 196/243 (80.7%) at off study• 359/495 patients (72.5%) felt the ePRO data facilitated discussions with their care team at 3 months and 188/244 (77.0%) at off study• 381/494 (77.1%) stated that providing their ePRO data made them feel more in control of their own care at 3 months, which increased to 205/244 (84.0%) at off study• 47 (83.9%) nurses indicated that ePRO data improved the quality and efficiency of their discussions with patients
de Jong et al.^10^	Outpatient, Netherlands	Pragmatic RCT at four hospitals	Patients with IBD without an ileoanal or ileorectal pouch anastomosis	Participants in the intervention arm (n = 465) used the system for 12 months and were instructed to plan at least one routine outpatient visit per year Additional follow-up visits were scheduled based on symptom alerts or at the requests of individual patients	Patients in standard care group (n = 444) continued their routine follow-up visits, with opportunity to schedule an extra visit if symptoms relapsed. At baseline and after 12 months, all participants received a paper questionnaire regarding perceived quality of care, medication adherence, quality of life, self-efficacy, disease-related and medication-related knowledge and smoking behaviour	Primary: number of outpatient visits and patient-reported quality of care Secondary: adherence to treatment, quality of life, self-efficacy, disease-related and medication-related knowledge, smoking behaviour, disease outcomes (number of flares, emergency visits etc.)	When parameters recorded by the monitoring modules exceeded predefined thresholds, alerts (red flags) were created on the administrator page of each local hospital. The page was checked at least twice daily except on weekends. If an alert was received, clinical staff contacted the patient for further assessment within two working days. Visits to the outpatient clinics were based on the nature and severity of clinical complaints. At any time, patients were able to contact the medical teams by messaging the administration offices	• At 12 months, the mean number of outpatient visits to the gastroenterologist was significantly lower in the intervention (1.26 (SD 1.18)) than in the standard care group (1.98 (SD 1.19)); estimated intervention effect –0.72 (95% CI –0.87 to –0.56); *p* < 0.0001• Outpatient visits to the nurse did not differ significantly between groups• The mean number of telephone consultations with the gastroenterologist was significantly lower in the intervention- than in the standard care group, but the mean number of telephone consultations with the nurse did not differ significantly• Patients in the intervention and standard care groups reported similar and high scores for quality of care at 12 months (8.16 (SD 1.37) vs. 8.27 (1.28), respectively • The mean numbers of flares, courses of corticosteroid treatment, emergency visits and IBD-related surgeries did not differ significantly between the two groups• The mean number of hospital admissions was significantly lower in the intervention- than in the standard care group (16 vs. 29); estimated intervention effect –0.05 (95% CI –0.10 to 0.00); *p* = 0.046)• Adherence to medication at the end of the trial was significantly higher in the intervention group than in the standard care group• Both groups reported normal values for quality of life and high scores for self-efficacy, disease-related and medication-related knowledge, with no significant differences between groups
de Thurah et al.^11^	Outpatient, Denmark	Pragmatic non-inferiority RCT at two centres	Patients with RA	AmbuFlex ePRO system used as a decision aid in deciding whether patients required an outpatient appointment. The 11-item Flare-RA was used Arm 1: Rheumatologist follow-up (PRO-TR) (n = 93)Arm 2: Follow-up by a nurse (PRO-TN) (n = 88)	Physicians saw patients in the outpatient clinic every 3–4 months (n = 94)	Primary: change in DAS28 after week 52 Secondary: physical function, quality of life and self-efficacy	ePRO telehealth for disease control. Patients in the AmbuFlex groups were seen in outpatient clinic if their Flare-RA score was ≥2.5 and/or their C-reactive protein (CRP) level was ≥10 mg/l	• Non-inferiority was established for the DAS28 in both AmbuFlex groups when compared to usual care. In the ITT analysis: • mean differences in the DAS28 score between PRO-TR versus usual care = −0.10 ((90% CI): −0.30, 0.13)• Between PRO-TN versus usual care = −0.19 (90% CI: −0.41, 0.02) • Including one yearly visit to the outpatient clinic patients in: • PRO-TN: 1.72 ± 1.03 visits/year• PRO-TR: 1.75 ± 1.03 visits/year• Usual care: 4.15 ± 1.0 visits/year
Efficace et al.^12^	Outpatient, Italy	Online survey of clinician perceptions of the utility of GIMEMA-ALLIANCE ePRO Platform	Patients diagnosed with any haematologic malignancy	180 of 201 (90%) patients were signed up for the study. Twenty-three haematologists completed the experience survey. Only one was involved in platform development	Not applicable	Physician experience of ePRO platform over 6 months	Remote ePRO monitoring. Based on a pre-defined algorithm, alerts are sent to medical staff in the presence of clinically important problems, symptoms or problems with adherence to therapyGraphically displayed PRO results were available to patients and physicians	• 16 physicians (69.6%) agreed that using the platform helped them to better understand their patients’ general health status and symptoms • 21 (91.3%) agreed/strongly agreed that the ePRO data facilitated accurate documentation of patients’ symptomatic AEs• 82.6% and 60.9% felt that the ePRO data assisted with the identification of low-grade and high-grade symptomatic AEs, respectively• 20 haematologists (87.0%) considered the ePRO data useful in setting up unplanned visits with patients • Upon receiving alerts of clinically important problems/symptoms, 14 (60.9%) physicians accessed the clinical portal the same day and 16 (69.6%) phoned their patients.18 haematologists, 7 (30%) often or 6 (26%) very often used the ePRO data for their discussions with the patients. However, 7 (30%) sometimes, 8 (34.8%) rarely, 4 (17%) never used the information for discussions with other colleagues • 20 (87%) haematologists agreed or strongly agreed that ePRO data enhanced their communication with patients, and 21 (91.3%) agreed that the data were useful for patient management and shared decision-making. All considered ePRO data useful in suggesting supportive care strategies
Girgis et al.^13^	Outpatient, Australia	Mixed‑methods (survey and qualitative interviews) evaluation of ePRO implementation	Patients with lung cancer	n = 48 patients	63 patients receiving standard care	• Impact of ePROs on the number of emergency admissions • Impact of ePROs on the use of the cancer assessment unit • Impact of ePROs on referral rates	Intended to support patient management. ePRO results were reviewed by care coordinators. Tailored self-management resources were provided Interventions or referrals were offered in response to alerts generated for patients with high levels of psychosocial distress or physical symptoms	• Patients in the ePRO group had significantly fewer visits to the cancer assessment unit for problematic symptoms (*p = *0.035)• ePRO assessments resulted in 146 referrals to allied health services, most frequently for social work, dietetics, physiotherapy and occupational • Patients in the ePRO group were more likely to be offered referrals than those in the control group (71% vs. 29%, *p* < 0.0001) • No between-group differences in ≥ 1 ED presentation (multivariable logistic regression; ePRO 58.3%, controls 60.3%)
Haverman et al.^14^	Paediatric outpatient, Netherlands	Cohort intervention study	Children with JIA	Children or parents (n = 176) completed online questionnaires before outpatient consultations	All patients were initially in a control period	• Differences in HRQoL scores between control and intervention• Effect of the intervention• Satisfaction	To inform consultations with paediatric rheumatologist (support care within outpatient visit). The study was divided into control period, where ePRO data were not provided to consultants, and intervention period, where data were provided and discussed during consultation	• The use of ePRO data increased discussion of psychosocial topics (*p* < 0.01); however, it did not affect referrals to a psychologist or parental satisfaction with care • Parents and physicians evaluated the use of the ePRO as positive in 80% to 100% of the consultations
Laurberg et al.^15^	Outpatient, Denmark	RCT, pragmatic non-inferiority design	Patients with type 1 diabetes	160 patients in the DiabetesFlex (flexible AmbuFlex-based follow-up) arm completed their questionnaire 2 weeks before consultation. They had an initial in-person consultation with clinicians	160 patients were on standard care, i.e. fixed healthcare provider-initiated face-to-face consultation every 4 months	• Primary: mean change in HbA1c from baseline to 15 months • Secondary: blood pressure, lipid levels, frequency of visits, WHO-5 score, PAID scale and experience of participation in own care	Manage outpatient appointments. Patients had options of in-person visit, telephone consultations or cancelling their appointment for the last two annual consultations. A nurse evaluated responses ensuring that they were clinically safe to change or cancelled a consultation	• Adjusted mean difference in HbA1c between both arms was similar and below the predefined non-inferiority margin • No intergroup mean changes in lipid or blood pressure were observed • No patients were hospitalised with severe hypoglycaemia or ketosis/ketoacidosis during the 15-month period • DiabetesFlex participants had increased mean WHO-5 index of 4.5 (1.3, 7.3), participation score of 1.1 (0.5, 2.0) and decreased PAID score of −4.8 (−7.1, −2.6)• Patients in the DiabetesFlex arm changed 23% of their face-to-face appointments to telephone consultations, cancelled more visits (17% vs. 9%) and stayed away without cancellation less often (2% vs. 8%)• The specialist nurse judged unsafe in four instances the decision by patients in the DiabetesFlex arm to cancel or change their appointments• After study end, 54% of controls opted to switch to DiabetesFlex and 94% in the DiabetesFlex group opted to stay on that plan
Richter et al.^16^	Outpatient. Germany	Proof-of-concept study for the RheumaLive App	Patients with RA	60 patients completed FFbH, and RADAI in paper and via the RheumaLive App on the clinic’s smartphone at baseline. 51 subsequently filled in paper and ePROMs at home	Not applicable	• Measurement equivalence of ePRO to paper • Patients’ capability to enter data in the smartphone App• Retention rates• User experience• Preferences of patients and physicians	Electronic diary and PROs for RA patients, remote and in clinic to support care. Patients were followed-up approximately every 3 months for 9 months Algorithms calculated questionnaires’ scores	• Of the 22 patients that were asked for their opinions of the App at 3 months, 16 stated that providing their ePROs had a ‘partial influence’ on patient–physician interactions during outpatient visits • 63.2% of patients reported that it was easier or partially easier to document disease course using the app • This influence was considered positive by 10 patients and physicians suggested that the use of the App facilitated adherence to therapy in seven patients
Riis et al.^17^	Outpatient, Denmark	Pilot RCT	Patients treated for early breast cancer	All patients completed online PRO questionnaires (Patient Experience Questionnaire, EORTC QLQ-C30, the EORTC breast cancer module (QLQ-BR23) and three questions on unmet needs). However, the ePRO data were only available to the PI (a clinician) for patients in the ePRO-based follow-up (intervention) arm (n = 60)	64 patients received standard follow-up care with prescheduled consultations every 6 months (total of 4)	• Primary: satisfaction with assigned follow-up care and unmet needs measured every 3rd month during 2-year period• Secondary outcomes: use of consultations, adherence to treatment at 2 years, and quality of life measured every 3rd month during a 2-year period	Patients in the ePRO-based follow-up group could request clinical consultation after completing their ePROs. If they indicated serious side effects or symptoms, but did not request a consultation, the PI contacted them to discuss	• Women in the standard care group attended twice as many consultations during the 2-year follow-up period compared to those in the ePRO-based follow-up group; 4.3 (95% CI 3.9–4.7) versus 2.1 (95% CI: 1.6–2.6), *p* < 0.001 • There were no statistically significant differences in patients’ satisfaction, unmet needs, quality of life or adherence to treatment
Samuel et al.^18^	Outpatient, USA	Sequential explanatory mixed methods research design (satisfaction surveys and interviews)	Patients with bladder and prostate cancer	The ePRO questionnaire comprised PROMIS short forms and the Bladder Cancer Index or Expanded Prostate Cancer Index Composite and was completed prior to an in-clinic appointment (n = 30 black; 49 white)	Not applicable	Racial differences in experiences of using an ePRO system among a cohort of black and white patients	A symptom report that summarised symptom severity longitudinally was provided to patients and their clinicians during clinic appointments	• Most participants selected the web-based option (77.7% black; 96.0% white) • Black patients were more likely to use the automated telephone-based system • ePRO system was generally perceived as very helpful/helpful in aiding symptom recall (77.8% black; 76.0% white)• White patients valued it more for facilitating a better understanding of their symptoms and enabling symptom tracking, while black patients valued the ePRO system more for facilitating symptom-related discussions and improving communication with clinicians (88.9% black; 56.0% white)
Schick-Makaroff et al.^19^	Home dialysis clinics, Canada	Longitudinal qualitative research design	Patients on dialysis	Patients were invited to attend their regularly scheduled clinic 15 min early to complete ePROs before their appointment. They completed electronic versions of ESAS and KDQOL-36 before their clinic appointment (n = 99). Nurses also participated (n = 12). Data were collected on the ePRO items that were discussed during the consultations, who initiated the discussions, and how often the nurses referenced the ePRO data	Not applicable	• The extent ePRO data were used in clinical encounters over 6 months• Actions taken in response to ePRO data over 6 months	PRO results were given to healthcare providers. Patients were offered their reports, and KDQOL patient education materials tailored to their results. Nurses saw patients first, along with the ePRO data. Researchers joined patients and nurses in clinic rooms to observe use of ePRO data. All interactions were audio recorded	• In 62 (38%) interactions, where discussions were triggered by patients’ ePRO data, the nurses made no changes to the patients’ care plan • The most frequent actions taken by the nurses based on the ePRO data included referrals or recommendations for follow-up with another healthcare professional, further assessments, requests for blood tests, recommendations to wait and monitor symptoms, and patient education • Of 165 nurse–patient interactions, nurses used the ePRO data to initiate discussions and clarify symptoms through focused assessments during 103 interactions • In 40 (24%) interactions, the nurses reviewed the ePRO data before starting their general assessment, while the rest of the time, they integrated elements of the ePROs into their regular practice• In 32 (19%) interactions, the nurses used the ePRO data to prioritise or steer the discussions• The ePRO data prompted the discussion of 24 specific issues. The most frequently prompted issues were itchiness, appetite, problems with sleeping, tiredness and shortness of breath. Discussions about sexual function and physical appearance were only initiated by patients
Schougaard et al.^20^	Outpatient, Denmark	Multi-centre, observational implementation study	Patients with epilepsy, coronary heart disease, narcolepsy, sleep apnoea, prostate cancer, asthma, RA, colorectal cancer and renal failure	AmbuFlex system, where the patients’ ePROs determined the need of an outpatient consultation	Not relevant, as system has been implemented for routine use	Impact on utilisation of resources in epilepsy, sleep apnoea and prostate cancer clinics	A traffic light alert system was developed. Green: No need of contact Yellow: May need of contact. A clinician decides whether further contact is needed. Red: Definite need of contact or the patient asks for a consultation	• Of 8256 remote PRO responses from epilepsy outpatients, up to 48% were handled without further contact • For sleep apnoea, up to 57% of the 1424 remote PRO responses did not require further contact • For prostate cancer, up to 26% of the 347 remote PRO responses did not require further contact• Completion rates were over 90% at baseline and follow-up
Schougaard et al.^21^	Outpatient, Denmark	Parallel two-arm pragmatic RCT	Patients with epilepsy on fixed-interval PRO-based follow-up with web-based questionnaires	Patients (n = 346) on patient-initiated PRO-based follow-up (open access telePRO)	Patients (n = 247) on fixed-interval PRO-based follow-up (standard telePRO)	• Primary: number of outpatient contacts from a regional registry, admissions, emergency room visits • Secondary: self-reported outcomes namely general health, well-being, health literacy, self-efficacy, number of seizures, side effects, safety and satisfaction	Patients in open access arm were asked to contact the outpatient clinic when they felt it necessary. Thus, at any time during the follow-up period, patients could indicate a need for contact with the outpatient clinic by filling in the disease-specific questionnaire	• No significant differences in the use of healthcare resources, patient self-management or satisfaction in the patient-initiated PRO-based initiative compared to fixed-interval PRO-based follow-up
Seppen et al.^22^	Outpatient, secondary rheumatology care centre, Netherlands	Non-inferiority RCT	Patients with RA who have low disease activity and without treatment changes in the past 6 months	Patients (n = 49), in the intervention group carried out weekly self-monitoring by completing RAPID3, in a smartphone app designed for this purpose) with a single pre-planned consultation at 12 months Information about patient-initiated care was provided, patients were told they could contact the outpatient clinic when they deemed it necessary (in case of questions or symptoms)	In the usual care group (n = 53), pre-planned outpatient clinic visits were continued at the discretion of the treating rheumatologist (usually every 3 to 6 months). Patients in the usual care group were also allowed to contact the outpatient clinic if necessary	• Co-primary: non-inferiority in terms of change in DAS28-ESR score after 12 months and the ratio of the mean number of consultations with rheumatologists between the groups • Secondary: The number of consultations (by telephone and in person) with nurses; patient empowerment (EC-17); patient–physician interaction (PEPPI-5); patient compliance (CQR-5); patient satisfaction with treatment (TSQM-9); patient satisfaction with healthcare (on three domains: ease of contacting our hospital, satisfaction with healthcare received, and likelihood of recommending hospital); physician satisfaction; usability of the application (SUS)	The results of the RAPID3 were used for self-monitoring by patients during the year, to reflect on the course of their disease, and to contact the outpatient clinic in a timely manner in case of progressive complaints Additionally, the physician or nurse at the outpatient clinic had access to their data to improve communication. In the app, a RAPID3 algorithm was used to identify potential RA flares. Flare notifications informed patients about possible flare, linked to self-management tips, and advised patients to contact a rheumatology nurse if deemed necessary by the patient. Scores in the app were not used to trigger contact from clinician to patient. Data collected in the app were synchronised in real time with the EMR	• DAS28-ESR slightly increased in both groups (ΔDAS28-ESR in app intervention group was 0.27 vs. 0.35 in usual care group) • Non-inferiority was established, as the 95% CI of the mean difference in DAS28-ESR between the groups was within the non-inferiority limit • The total number of outpatient visits with nurses was also lower in the app intervention group • Proportion of in-person rheumatologist consultations to total number of rheumatologist consultations (both in-person and telephone consultations) changed pre- and post-COVID-19 (after 1 March 2020). In the intervention group, the proportions changed from 0.15 (3 of 20) to 0.20 (13 of 65), and in the control group, the proportions changed from 0.59 (22 of 37) to 0.30 (34 of 112). This suggests that the control group would likely have had more in-person consultations without COVID-19 and fewer telephone consultations • Number of flare visits was 12 (in 11 patients) in the app intervention group and 18 (in 11 patients) in the control group. These consultations led to an intensification of treatment with DMARDS or steroids in nine patients in both groups. For the app intervention group, 8 of 12 flare consultations were not preceded by a flare notification. During the study, there were 40 flare notifications, of which 36 did not lead to a consultation (10% of the prompts led to a consultation) • There were no significant differences between groups in RAPID3, EC-17, PEPPI-5 or CQR-5 at 12 months • Satisfaction with healthcare was high in both groups and was not statistically different • Usability was rated good/excellent
Seppen et al.^23^	Outpatient, Netherlands	Medical records review	Patients with RA who have low disease activity scores	Patients completed RAPID3, PASS and a flare question prior to their clinic appointments over 3 months. 321 records included were completed by 302 patients (19 patients with 2 entries)	Not applicable	• Primary: PPV of having a low disease activity score on an ePRO for not receiving a DMARD or steroid intensifications within 2 weeks • Secondary: Combination of ePROs that give the best PPV	Not applicable	• RAPID3, PASS and flare question had a high diagnostic accuracy to identify individuals who will not receive a DMARD or steroid intensification in the following 2 weeks • The combination of the RAPID3 and flare question yielded the best combination of diagnostic accuracy and highest percentage of patients who could be eligible to skip an outpatient clinic visit
Short et al.^24^	Outpatient, USA	Feasibility study at 2 sites (PROgress study)	People with HIV (PWH)	1630 PWH completed PROs. Acceptability E-scale was completed by 1102 PWH. Where possible, skip logic was applied to reduce respondent burden. Eligibility for future follow-up assessments within the PRO platform was set for a minimum of 105 days, to minimise potential clinic burden and/or patient survey fatigue	Not applicable	5 outcome domains: reach, effectiveness, adoption, implementation and maintenance (RE-AIM) + acceptability. The primary objectives of the study were to (1) understand and assess the number, proportion and representativeness of individuals who are willing and able to successfully engage in the process (reach); (2) to assess the impact of the PRO intervention on the patient–provider interaction, clinic operation and clinical/medical practice(effectiveness); (3) to assess the number, proportion and representativeness of clinic personnel adopting the intervention (adoption); (4) to assess the degree to which the fidelity of PRO integration is upheld, including consistency of delivery, use as intended and the time and cost of the intervention (implementation); (5) to assess the extent to which the intervention can be sustained over the longer term at the setting and individual levels (maintenance); and (6) to assess the acceptability of the intervention from the perspective of patients, providers and other clinic staff	Completed PROs were scored using automated algorithms. Results were summarised in a report that was shared with the provider immediately before the clinic visit. Comparison of chart reviews from the preparation phase (without PRO feedback to providers) and the delivery phase (with PRO feedback to providers, namely doctors, nurses, physician assistant, pharmacists)	• When providers received ePRO data before clinic appointments, they were more likely to document suicidal ideation (*p* = 0.002), anxiety (*p* < 0.001), and refer PWH to mental health services for anxiety (*p* = 0.008) • Chart review showed that provision of ePRO data to providers improved patient–provider communication and increased the number of complex health and behavioural issues identified, recorded, and acted on• These issues included suicidal ideation (88% with ePRO feedback vs. 38% without) and anxiety (54% with ePRO feedback vs. 24% without)• Although 22 (12%) of the 181 PWH who declined/failed to complete ePROs, PWH thought ePRO assessments were unnecessary/not useful, post-clinic surveys indicated that 82% of PWH who completed ePROs and providers felt the use of ePRO added value to the clinic appointments.• Of the 200 PWH who completed the post-clinic survey, 88% agreed or strongly agreed that ePRO assessments helped them consider overall health, recall health concerns to raise (80%), discuss topics that might otherwise not have arisen (76%), discuss sensitive issues (71%) and decide what to talk about (67%).• 9 providers (82%) either agreed or strongly agreed that ePRO data helped prioritise discussion points, identified issues that might not have been raised, and encouraged discussions of sensitive topics.
Zhang et al.^25^	Tertiary care hospitals, China	Open label multicentre RCT	Patients on cancer immunotherapy	Using the ePRO App, patients (n = 141) completed weekly questionnaires and uploaded pictures of examination results between visits	Researchers educated patients (n = 137) and their caregivers about immunotherapy and common symptoms of irAEs at baseline. Patients had standard follow-up including clinic visits every 21 days and via the telephone every 3 months. Patients could visit the clinic when they felt any discomfort	Incidence of serious (grades 3 to 4) irAEs, emergency department visits, QOL, time spent implementing the ePRO model, rate of treatment discontinuation and death	Patients received standardised advice via the app for grade 1 or 2 irAEs. For grade 3 or 4 irAEs, alerts were sent to the healthcare team for immediate assessment and intervention	• The ePRO group showed a reduced incidence of serious irAEs (29 of 141 (20.6%) vs. 46 of 137 (33.6%); hazard ratio (HR), 0.51 (95%CI, 0.30–0.88); *p = *0.01) • Fewer ED visits (23 of 141 (16.3%) vs. 41 of 137 (29.9%); HR, 0.46 (95%CI, 0.26–0.81); *p = *0.01) • A lower rate of treatment discontinuation (5 of 141 (3.6%) vs. 15 of 137 (11.0%); HR, 0.30 (95%CI, 0.11–0.85) *p = *0.02) • A higher QOL (mean (SD) score, 74.2 (15.1; 95%CI, 71.7-76.9) vs. 64.7 (28.5; 95%CI, 61.0–68.4); *p = *.001) • Less time implementing follow-up (mean (SD), 8.2 (3.9; 95%CI, 5.0–10.6) minutes vs. 36.1 (15.3; 95%CI, 33.6–38.8) min; *p* < 0.001)

irAE: immune-related adverse events; ePROM: electronic patient-reported outcome measure; QOL: quality of life; RA: rheumatoid arthritis; RCT, randomised controlled trial.

### Reduction in outpatient appointments

Ambuflex, a generic ePRO system, is currently being used in Denmark for outpatient management of patients across several chronic conditions.^26^ Use of the system has led to substantial reductions in outpatient appointments of 48% (epilepsy), 57% (sleep apnoea),^20^ 45% (inflammatory bowel disease [IBD] with mild or no disease activity),^8^ 2.4 fewer extra visits per year (RA)^11^ and a 23% change of face-to-face appointments to telephone consultations (type 1 diabetes).^15^ However, socioeconomically disadvantaged patients with epilepsy may be less likely to be referred for remote outpatient follow-up.^27^ Evidence suggests no differences between flexible patient-initiated PRO-based follow-up and fixed-interval PRO-based follow-up in the use of healthcare resources, patient self-management or satisfaction.^21^

Findings from studies of other ePRO systems included significantly fewer in-person visits for problematic symptoms in patients with lung cancer^13^; reduced outpatient visits and hospital admissions in patients with IBD^10^; a decrease in the need for follow-up visits after breast reconstruction surgery^28^; fewer consultations in patients treated for early breast cancer on ePRO-based follow-up^17^; approximately 28 min lower mean time requirement for implementing follow-up in patients on cancer immunotherapy^25^; and the potential to predict which patients with RA (who have low disease activity scores) can skip a clinic visit and those who may not require certain medication for 2 weeks after providing their ePROs.^23^

### Better understanding and documentation of patients’ symptoms

The use of ePROs led to a significant difference of 13% in incidence of serious immune-related adverse events (irAEs), 7.4% lower rates of treatment discontinuation and better quality of life in patients on cancer immunotherapy.^25^ Clinicians reported that ePRO data facilitated their understanding of patients’ general health status and symptoms,^12^ identification of low-grade and high-grade symptomatic adverse events,^12^ accurate documentation of patients’ symptoms^9,12,24^ and improved patient adherence to treatment.^10,16,29^

### Initiation of timely interventions

Various studies described clinician responses to ePRO data, which suggest timely/earlier intervention when symptoms of clinical concern are present.^30,31^ These included: telephoning patients upon receiving ePRO alerts of clinically important symptoms^12^; referrals to other healthcare professionals^19^; mental health services^24^; further assessments; requests for blood tests; recommendations to wait and monitor symptoms; and patient education.^19^ Clinicians also stated that ePRO data were useful in the clinical management of patients and shared decision-making.^12^

### Facilitation of patient–clinician interactions/communication

Several studies reported that the use of ePRO data facilitated patient–clinician interactions.^9,12,14,16^ Specifically, ePRO data were used to initiate discussions including those around complex health and behavioural issues such as suicidal ideation and anxiety in people with HIV,^24^ and clarify symptoms through focused assessments during clinical appointments.^19^ Clinicians may review the ePRO data before starting their general assessment or integrate elements of the ePROs into their regular practice.^19^ For example, a recent implementation of a co-designed ePRO dashboard for IBD reported that it helped focus discussions on what mattered most to the patient; use of this dashboard during the clinic visit versus outside the visit was also associated with a twofold increase in shared decision-making.^32^ While clinicians often initiated the discussion of various issues highlighted by the ePRO data,^33^ there may be some reluctance in initiating discussions about sensitive issues such as sexual function and physical appearance.^19^

Patients confirmed that providing ePROs helped them consider their overall health, recall health concerns to raise,^18,24^ discuss topics that might otherwise not have arisen, discuss sensitive issues, decide what to talk about and feel more in control of their own care.^9,24,34^ There might be racial differences in the benefits patients derive from the use of ePROs.^18^ A study reported that white patients valued the ePRO system for facilitating a better understanding of their symptoms and enabling symptom tracking, while black patients valued the ePRO system more for facilitating symptom-related discussions and improving communication with clinicians.^18^

## Barriers to implementation and potential solutions

Several barriers to implementation were identified as outlined below.

### Inclusive, equitable data collection

Low levels of computer and health literacy or challenges with language have limited implementation within certain groups, predominantly black and non-English-speaking populations.^13,18^ Patients from socioeconomically disadvantaged backgrounds may be less likely to be considered for ePRO-based follow-up.^27^ Therefore, the successful implementation of ePROs in clinical care could exacerbate health disparities if the advantages of ePRO are not also available to already disadvantaged patients. Interestingly, it has been reported that computer-inexperienced patients may derive greater benefit from ePRO monitoring in terms of emergency department visits, hospitalisation and survival compared to computer-experienced patients.^31^ Similarly, the use of ePROs in outpatient care may be more beneficial for black patients in improving patient–clinician communication.^18^

These findings demonstrate why it is crucial that the use of ePROs for outpatient care is carefully considered, planned and implemented to ensure that people from underserved populations are not further disadvantaged.^35^ Ways to promote inclusive and equitable and inclusive completion have recently been described, including co-designing systems with user input, considering the needs of the target population such as clinical characteristics, cultural and language needs, literacy and health literacy and considering ways to promote digital inclusion.^35^ User-centred design may also help promote adherence to completion of ePROs. Digital inclusion and adherence could be promoted by providing alternative modes of delivery, for example, bring your own device, provision of device, web completion or voice response systems (that do not require internet access), phone calls from staff and the options of remote or in-clinic PRO provision.^35^

### Impact on clinical workflow

Concerns have been expressed that using ePROs for routine patient management could potentially increase clinical workload and negatively impact on workflow if clinicians are uncertain about the benefits of ePRO, are not clear how to integrate ePRO into their practice, do not find PROs clinically actionable and are not consulted on how to build ePRO into their workflow.^33,36–39^ However, a survey of clinicians who participated in the PROgress study showed that generally the implementation of ePROs was perceived as having a minimal and/or manageable impact on providers’ workload. The clinicians were not unanimous on whether the use of ePROs saved time during consultations.^24^ Co-development of systems with clinical input may help ensure that systems meet end user needs; however, further work is required to assess the impact of systems on workflow and workloads.

### Cost and cost-effectiveness

The issues of cost and cost-effectiveness could significantly influence the decision by healthcare providers to commission the development and implementation of ePRO systems.^40^ A recent review showed that in the long run, ePRO systems could be cost-effective when implemented for routine clinical practice.^40^ However, there is a need for studies to evaluate cost-effectiveness specifically in outpatient contexts. There are some important requirements with cost implications that need to be considered early during the planning phase of implementation. For instance, many of the studies we found employed an algorithm to automatically analyse patients’ ePROs and flag concerning issues for attention removing the need for clinicians to review every assessment. Automatic reminders and training (patient and/or clinician) were provided by several studies to enhance completion rates and uptake. Short et al. reported that for in-clinic ePRO provision, on-site research coordinators required approximately 4 min per patient to explain the procedure, monitor ePRO completion and sanitise the tablet used (patients did not have the option of using their own devices).^24^

## Implications of findings

Our review provides evidence to support the implementation of ePRO systems for outpatient care. The studies reported improvements in healthcare resource utilisation including significant reductions in outpatient appointments without compromising patient outcomes or satisfaction with care. Stable patients who remotely provide their ePROs do not have to attend appointments unnecessarily and only those patients who really need to be seen in person are scheduled for face-to-face consultations. However, further studies will be required to evaluate the long-term impacts on patient safety and outcomes. It is also important to acknowledge that a proportion of patients, especially the elderly, may prefer face-to-face or telephone outpatient consultations regardless of their health status and may be concerned about or averse to the use of ePROs as a triaging tool. Brief telephone consultations in combination with ePRO reporting could be offered to stable patients.

Other potential benefits highlighted in this review include facilitation of the documentation and monitoring of patient symptoms, provision of timely interventions when required and improvement of patient–clinician interaction. While these may have positive effects on patient outcomes and satisfaction with the processes of care, there is an important concern that using ePROs for routine patient management could potentially increase clinical workload and negatively impact on workflow.^36–38^

ePROs may be implemented for flexible patient-initiated follow-up or for fixed-interval clinician-initiated follow-up. Only one study compared these approaches and there were no significant differences in healthcare resource utilisation or patient satisfaction.^21^ The authors suggested that patient-initiated PRO-based follow-up may be used as an alternative to fixed-interval PRO-based follow-up in patients who prefer this approach.^21^ The adoption of this approach will require the provision of appropriate patient education to ensure patient safety.

Finally, while several studies in this review confirmed that ePRO systems are highly acceptable to patients, some patients will choose not to engage. It should be noted that aside from the study by Schougaard et al.,^20^ the duration of the studies was between 3 and 24 months. Therefore, it is not certain that acceptability of the ePRO systems will remain at the same level in the long term.

## Looking ahead

Utilisation of ePROs for outpatient care could facilitate the tailoring of care to patient needs. Stable patients can be monitored remotely using ePROs, thereby avoiding unnecessary check-ups in outpatient clinics, associated costs such as travel and time off work, without lowering the quality of treatment. This efficient use of scarce healthcare resources could free up outpatient clinics for patients with high symptom burden/concerning symptoms, so they can be seen more quickly. With appropriate patient education, training and support, patients may potentially engage more in self-monitoring, management and shared decision-making if given access to their ePRO data. Other potential barriers that have been reported in literature can be appropriately addressed to facilitate implementation and uptake of ePROs.^41^

In all the studies, ePROs were used to inform clinical decision-making; however, as systems develop, wearables or blood tests could be used to supplement data collected via ePROs, and prediction models could be developed and refined using artificial intelligence to optimise triage approaches. However, these developments may lead to the classification of more ePRO systems as medical devices for regulatory purposes. Developers should ensure that systems are developed in accordance with medical device regulations for the jurisdiction of use. As medical devices, ePRO systems will be required to comply with stricter standards that may be challenging and influence their implementation and evaluation in clinical practice.

The utilisation of ePROs in the delivery of outpatient care could potentially allow a more responsive healthcare system, reduce demand for clinic appointments, reduce time to care with associated improved outcomes and enhance cost-effectiveness of healthcare delivery – all of which are beneficial for patients, their families and society.

## Declarations

### Guarantor

OLA.

### Provenance

Not commissioned; peer reviewed by Tamás Szakmany.

### ORCID iDs

Olalekan Lee Aiyegbusi https://orcid.org/0000-0001-9122-8251

Sarah E Hughes https://orcid.org/0000-0001-5656-1198

Melanie J Calvert https://orcid.org/0000-0002-1856-837X
